# Zimmer Natural Nail and ELOS nails in pertrochanteric fractures

**DOI:** 10.1186/s13018-021-02634-9

**Published:** 2021-08-18

**Authors:** Giuseppe Gargano, Nicola Poeta, Francesco Oliva, Filippo Migliorini, Nicola Maffulli

**Affiliations:** 1 Department of Trauma and Orthopaedic Surgery, AOU San Giovanni Di Dio E Ruggi D’Aragona, Via San Leonardo 1, 84131 Salerno, Italy; 2 Department of Medicine, Surgery and Dentistry, University of Salerno, Via S. Allende, 84081 Baronissi, SA Italy; 3 Department of Orthopedic, Trauma, and Reconstructive Surgery, RWTH University Hospital, 52074 Aachen, Germany; 4 Queen Mary University of London, Barts and the London School of Medicine and Dentistry, Centre for Sports and Exercise Medicine, Mile End Hospital, 275 Bancroft Road, London, E1 4DG England; 5 School of Pharmacy and Bioengineering, Keele University School of Medicine, Thornburrow Drive, Stoke on Trent, England

**Keywords:** Hip fracture, Pertrochanteric fracture, Intramedullary nail, ZNN, ELOS

## Abstract

**Background:**

Pertrochanteric fractures of the femur in the elderly are very common. As the average age of the population increases, the incidence of such fractures also raises, resulting in high healthcare costs. The type of surgical devices employed for their surgical management influences these costs.

**Methods:**

A comparative clinical study was conducted on patients operated by one single surgeon between December 2018 and November 2020 in a high-volume regional referral centre. All patients who received a Zimmer Natural Nail (ZNN) or ELOS devices were included.

**Results:**

In 119 (66.48%) of the 179 fractures, a ZNN nail was used. Post-operatively, the TAD (tip-to-apex distance) was measured at an average value of 17.05 (4.42–41.85) mm and the CalTAD (calcar-referenced TAD) at an average of 20.76 (10.82–43.63) mm. The mean hospitalization time was 10.19 (4–22) days. In the other 60 trochanteric fractures, an ELOS nail was used. Post-operative imaging indicated a TAD of 19.65 (5.08–31.4) mm and a CalTAD of 22.86 mm (12.66–33.77). The average time of the operation was 45.82 (20–110) min. The average period of hospitalization was 10.45 (5–24) days.

**Conclusion:**

Both devices give similar results in terms of short-term post-operative outcome and hospitalization. The price difference between the devices does not translate in different short-term results on the operated patients.

## Background

Hip fractures in the elderly are common, with an incidence of 250,000 cases per year in the USA and between 70,000 and 75,000 in the UK, resulting in significant healthcare costs [1]. Around 90% of hip fractures occur in individuals over 65 [2], and these fractures could reach 2.6 million by 2025 and 4.5 million by 2050 [3]. The mortality of patients after a hip fracture ranges from 10% in the first month to 30% in the first year. Mortality is however linked to the numerous comorbidities of these patients [3, 4].

Fractures that occur between the greater and lesser trochanters of the femur are defined as intertrochanteric fractures, and are extracapsular [5, 6]. The most widely used treatment for intertrochanteric fractures to date is internal fixation using sliding hip screws (SHS) or intramedullary (IM) devices [7, 8].

Baumgaertner et al. described the measurement of TAD (tip-to-apex distance) to evaluate the placement of a SHS within the femoral head [9]. The maximum distance to prevent device mobilization was determined to be 25 mm. Distances less than 25 mm were associated with no slippage of the cephalic screw [10]. In this context, “mobilization” refers to a post-operative sliding of the cephalic screw or of the device, leading to a change in the TAD greater than 3 mm [11]. Such event is more frequent with a TAD greater than 25 mm [12].

Kashigar et al. [13] related cephalic screw mobilization to the CalTAD (calcar-referenced TAD). The CalTAD is calculated in a similar fashion to the TAD in the lateral view, but differs in the antero-posterior (AP) view. In the AP view, the TAD follows an imaginary line passing through the apex of the cephalic screw, while in the CalTAD, the imaginary line parallel to the cephalic screw passes tangentially to the medial cortex of the femoral neck [14].

Intramedullary (i.e. cephalomedullary) devices have become very popular, as they have dedicated instrumentation which allows their precise insertion and guide the surgical procedure, positively influencing the duration of the procedure [15]. We compared two types of intramedullary devices routinely used in our department namely the Zimmer Natural Nail System (ZNN CephaloMedullary Femoral Nail Zimmer; Warsaw; IN, USA) and the ELOS (InTrauma, Rivoli, Italy) nail, studying the differences in both surgical and outcome terms of patients operated with these two devices. We also considered the cost of the device (ZNN or ELOS nail) and the length of hospital stay.

The primary purpose of this study was to investigate the difference in clinical outcomes and perioperative complications in patients operated with the two different devices. We hypothesised that the two devices promote similar outcomes when used by a trained surgeon. The secondary purpose was to clarify the potential challenges and pitfalls in implant positioning.

## Methods

Data on all patients operated in our department with the ZNN or ELOS devices by one single surgeon were collected in the period December 2018 to November 2020. There were 179 patients, of whom 40 were males and 139 females, with a mean age of 84.2 years (range 66 to 99 years). All patients suffered a pertrochanteric fracture, classified according to the AO/OTA system: 83 patients had a 31-A1 fracture, 73 had a 31-A2 fracture, and 23 had a 31-A3 fracture (Table 1).

**Table 1:** Patients included

	**Total**	**ZNN**	**ELOS**
**Age (median, range)**	84.2 (66–99) years; 7.19 SD	85.1 (66–99) years; 7.12 SD	82.9 (67–95) years; 7.08 SD
**Female/male**	119/40	93/26	46/14
**Type of fracture**	31-A1: 73	31-A1: 58	31-A1: 25
	31-A2: 83	31-A2: 49	31-A2: 24
	31-A3: 23	31-A3: 12	31-A3: 11

All the clinical data were stored in the files archived in the department. The results of laboratory investigations were stored in the computerized hospital database, which also contained the radiographic investigations performed before and after the surgery.

**Fig. 1:**
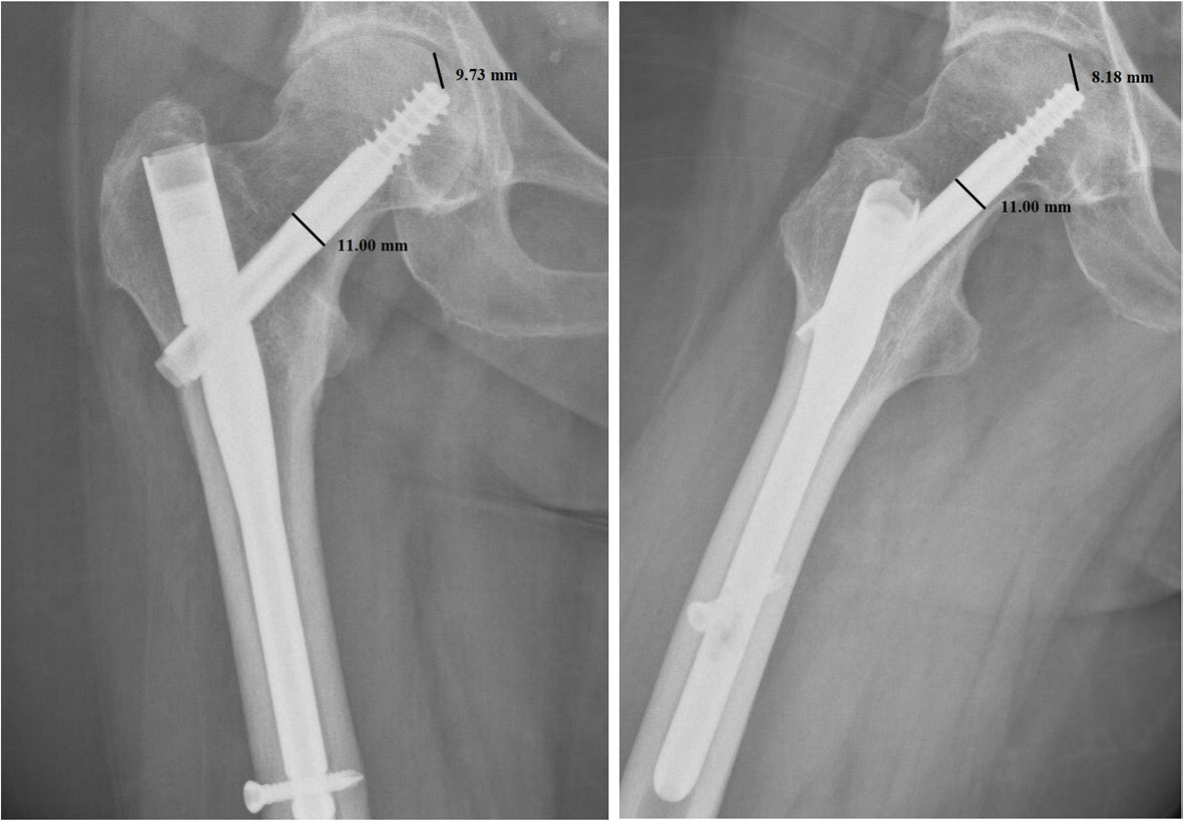
Measurements of tip-to-apex distance calculation in antero-posterior and lateral views

**Fig. 2:**
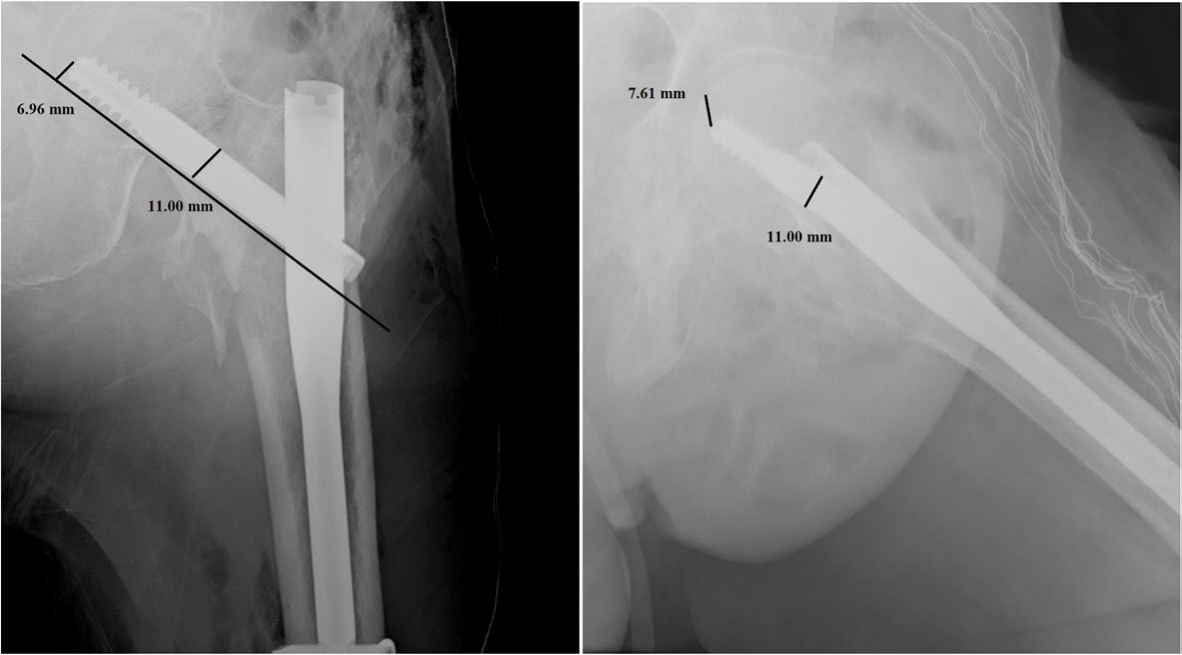
Measurements of calcar-referenced tip-to-apex distance calculation in antero-posterior and lateral views

Per each patient, we recorded age, sex, type of fracture, duration of surgery, transfusions performed, hospitalization time, TAD, CalTAD, haemoglobin variation, characteristics of the nail, and positioning of the same. All images were exported into the Surgimap Software (Nemaris Inc, New York, NY, USA) to measure the TAD and CalTAD, for each radiograph, knowing the diameter of the cephalic screw (Figs. 1 and 2). Each measurement per set of radiographs was repeated in a blinded fashion after 1 month in the same way comparing the two sets of measurements using Cohen’s Kappa test to calculate the intra-tester reliability.

Before surgery, patient signed an informed consent which detailed about the operative procedure, functional and cosmetic expectations, and possible complications related to the surgery, consenting also to be part of any outcome research.

All the surgical procedures were performed according to the manufacturer’s instructions [16, 17] by a fellowship-trained consultant orthopaedic surgeon who was fully familiar with both the nails and the instrumentation, being part of a departmental structure that performs a high number of such procedures a year. The surgeon had performed at least 50 procedures using each of the IM nails before starting this study.

### Choice of implant

The two devices used in the study (ELOS and ZNN) are the only ones with a single cephalic screw available in our centre. Both implants are certified for regular use in our country, and their materials have been certified and approved. Given the work flow in our setting, once it had been determined that a single cephalic screw intramedullary implant was appropriate, the nail actually used for a given patient was determined by the availability of the implant itself and was independent of the preference of the surgeon.

## Results

**Fig. 3:**
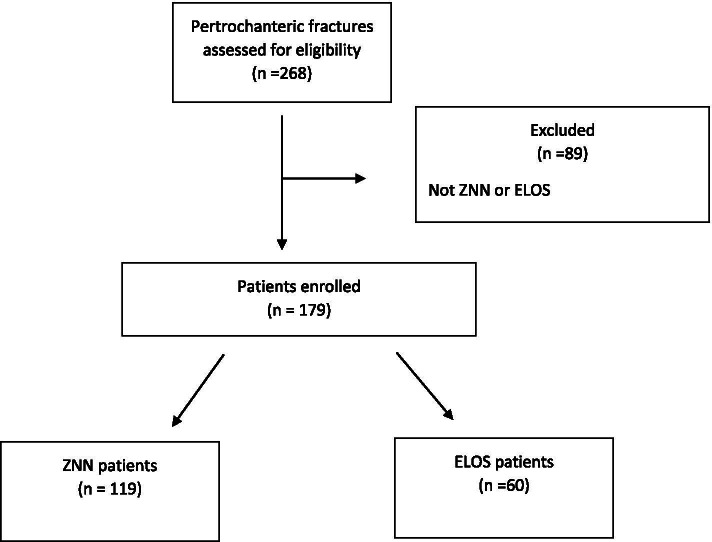
Work flow of patients

Patient data and fracture type data are described in Table 1, and the study results are described in Table 2. The characteristics of the patients enrolled in the study are described in Fig. 3.

**Table 2:** Results of the analysed data

	**ZNN**	**ELOS**	**Student’s *t* test**
**Duration of surgery**	41.76 (15–130) min; 17.2 SD	45.82 (20–110) min; 17.76 SD	*P* = 0.151
**Transfusions performed**	1.98 (0–6) U of blood; 1.43 SD	1.63 (0–5) U of blood; 1.46 SD	*P* = 0.350
**TAD**	17.50 (4.42–41.85) mm; 7.25 SD	19.65 (5.08–31.4) mm; 7.28 SD	*P* = 0.064
**CalTAD**	20.76 (10.82–43.63) mm; 5.58 SD	22.86 (12.66–33.77) mm; 5.75 SD	*P* = 0.027
**Hospitalization time**	10.19 (4–22) days; 3.44 SD	10.45 (5–24) days; 3.43 SD	*P* = 0.630
**Haemoglobin variation**	3.5 (0.3–6.6) g; 1.25 SD	3.25 (1.2–6.8) g; 1.28 SD	*P* = 0.209
**Nail positioning**	Cen-Ant: 4	Cen-Ant: 3	
	Cen-Cen: 43	Cen-Cen: 15	
	Cen-pos: 8	Cen-pos: 5	
	Inf-Cen: 10	Inf-Cen: 7	
	Inf-Pos: 48	Inf-Pos: 25	
	sup-ant: 4	sup-ant: 1	
	pos-sup: 0	pos-sup: 0	
	ant- inf: 0	ant- inf: 1	
	sup-cent: 2	sup-cent: 2	
**Costs**	700 euro	Short: 450 euro	
		Long: 520 euro

**Fig. 4:**
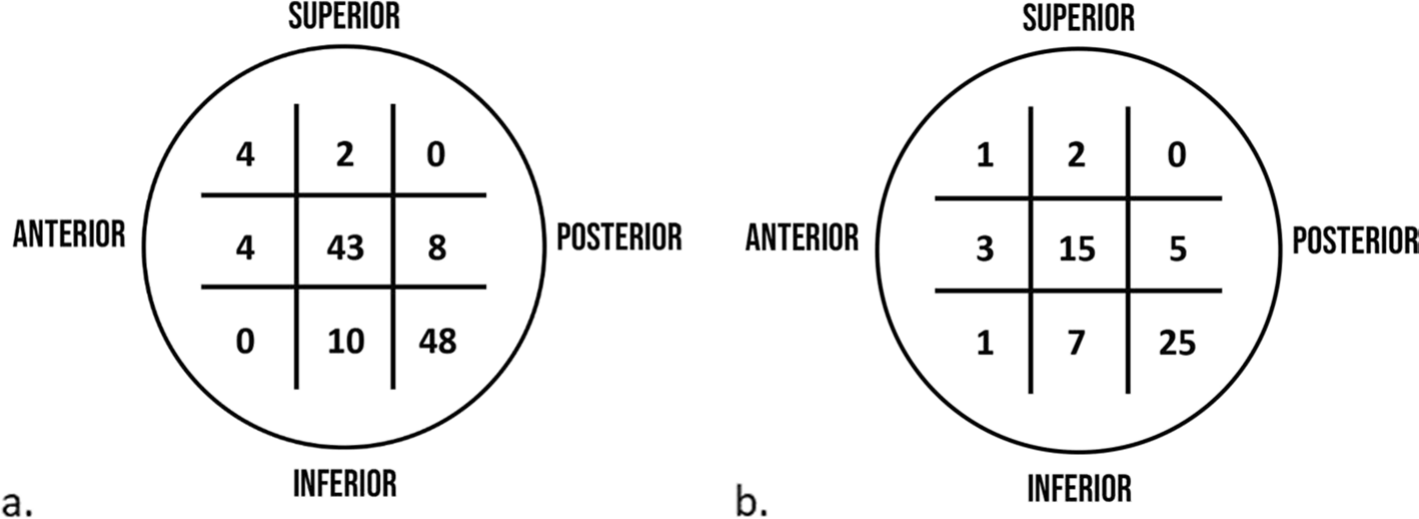
**a** Position of the cephalic screw in the head of the femur (Zimmer Natural Nail). **b** Position of the cephalic screw in the head of the femur (ELOS nail)

A ZNN nail was used in 119 (66.48%) of the 179 fractures, classified according to AO/OTA as 31-A1 in 58 patients, 31-A2 in 49 patients, and 31-A3 in 12 patients. The average duration of the surgery was 41.7 (15–130) min. Post-operatively, the TAD was measured at an average value of 17.05 (4.42–41.85) mm, and the CalTAD at an average of 20.76 (10.82–43.63) mm. The position of the cephalic screw in the head of the femur resulted inferior-posteriorly in 48 (40%) patients, in the centre of the head in 43 (36.1%) patients, inferior-centrally in 10 (8.4%) patients, central-posteriorly in 8 (6.72%) patients, centre-anteriorly in 4 (3.36%) patients, supero-anteriorly in 4 (3.36%) patients, and superior-centrally in 2 (1.68%) patients (Fig. 4a). The mean hospitalization time was 10.19 (4–22) days, during which a variation of Hb of 3.5 (0.3–6.6) g/dL was observed and an average of 1.98 (0–6) U of blood was transfused for patient.

An ELOS nail was used in the other 60 trochanteric fractures, classified as 31-A1 in 25 patients, 31-A2 in 24 patients, and 31-A3 in 11 patients. Post-operative radiographic parameters indicated a TAD of 19.65 (5.08–31.4) mm and a CalTAD of 22.86 (12.66–33.77) mm.

A TAD of 25 mm or lower was obtained in 80 (67%) of 119 patients in whom a ZNN device had been used and in 35 (58%) of 60 patients in whom an ELOS device had been used. A CalTAD of 25 mm or lower was obtained in 68 (57%) of 119 patients in whom a ZNN device had been used and in 30 (50%) patients in whom an ELOS device had been used.

The cephalic screw was located in the posterior-inferior area of the head in 25 (41.6%) patients, centre of the head in 15 (25%) patients, inferior-central in 7 (11.67%) patients, centre-posterior in 6 (10%) patients, centre-anterior in 3 (5%) patients, superior-centre in 2 (3.2%) patients, superior-anterior in 1 (1.6%) patient, and anterior-inferior in 1 (1.6%) patient (Fig. 4b). The average time of the operation was 45.82 (20–110) min. The average period of hospitalization was 10.45 (5–24) days, with a variation of haemoglobin during hospitalization of 3.25 (1.2–6.8) g/dL for which an average of 1.63 (0–5) U of blood were transferred for patient. The Student *t* test showed no significant differences between the two groups of patients.

Of the patients treated with ZNN, two had cut-outs and prosthetic surgery was performed.

One patient developed an infection, and the ZNN implant was removed. Two patients treated with an ELOS nail had infection but were treated pharmacologically. The reported infections were from gram-positive bacteria, possibly skin commensals. All other patients who returned to follow-up had a regular fracture healing process.

## Discussion

In the USA, over 90% of patients with proximal femur fractures are aged over 50 years. The incidence of such fractures is expected to double for every decade after age 50 [14], a significant health expenditure. In addition, elderly subjects often have comorbidities, and their health conditions are not optimal. This determines an increase in hospitalization time and difficulty in performing a second surgery if the index one fails [18]. After a hip operation, the 12-month mortality rate is estimated at 35% for men and 22% for women [19].

Modern IM nailing systems allow faster operating times than SHS devices, resulting in a reduction in intraoperative bleeding and earlier walking [20], but there is still the possibility of surgical failure [4]. Cut-out is the most frequent cause of surgical failure, ranging, in IM nailing, from 1.4 to 19%, depending on the type of fracture and device used [21].

**Fig. 5:**
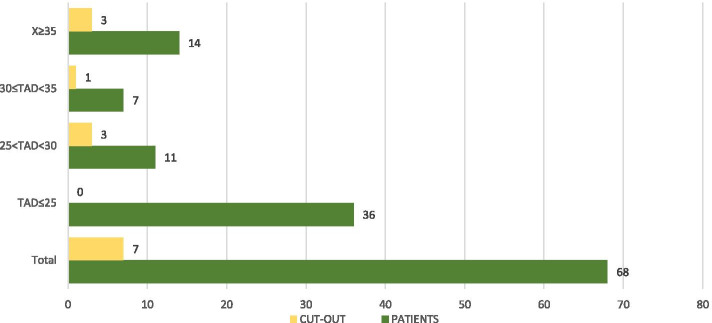
Cut-out in relation to tip-to-apex distance [14]

**Fig. 6:**
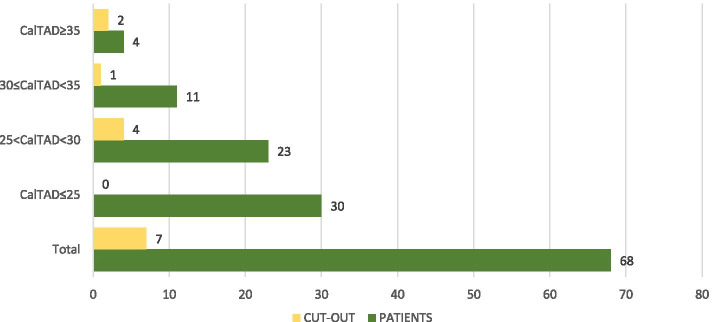
Cut-out in relation to calcar-referenced tip-to-apex distance [14]

The cut-out rate is higher if the cephalic screw is inserted into a posterior-inferior and anterior–superior location in the head: the central position of the cephalic screw is optimal in the lateral radiographic projection [9]. In the antero-posterior radiographic view, the central position of the cephalic screw is associated with a reduced incidence of cut-out. The centre of the head has a high bone volume that allows a better anchorage of the screw and is less affected by small movements of the device (Figs. 5 and 6) [14].

**Fig. 7:**
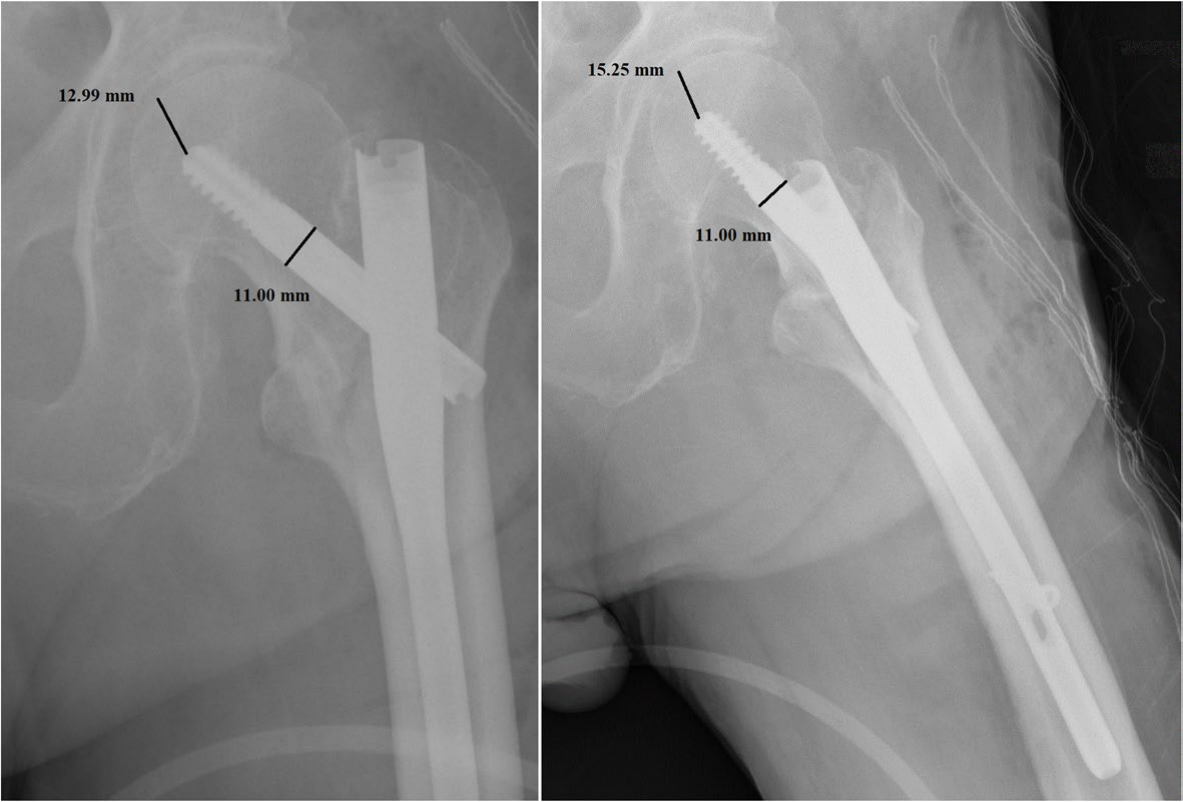
Measurements in antero-posterior and lateral; tip-to-apex distance > 25 mm (Zimmer Natural Nail)

**Fig. 8:**
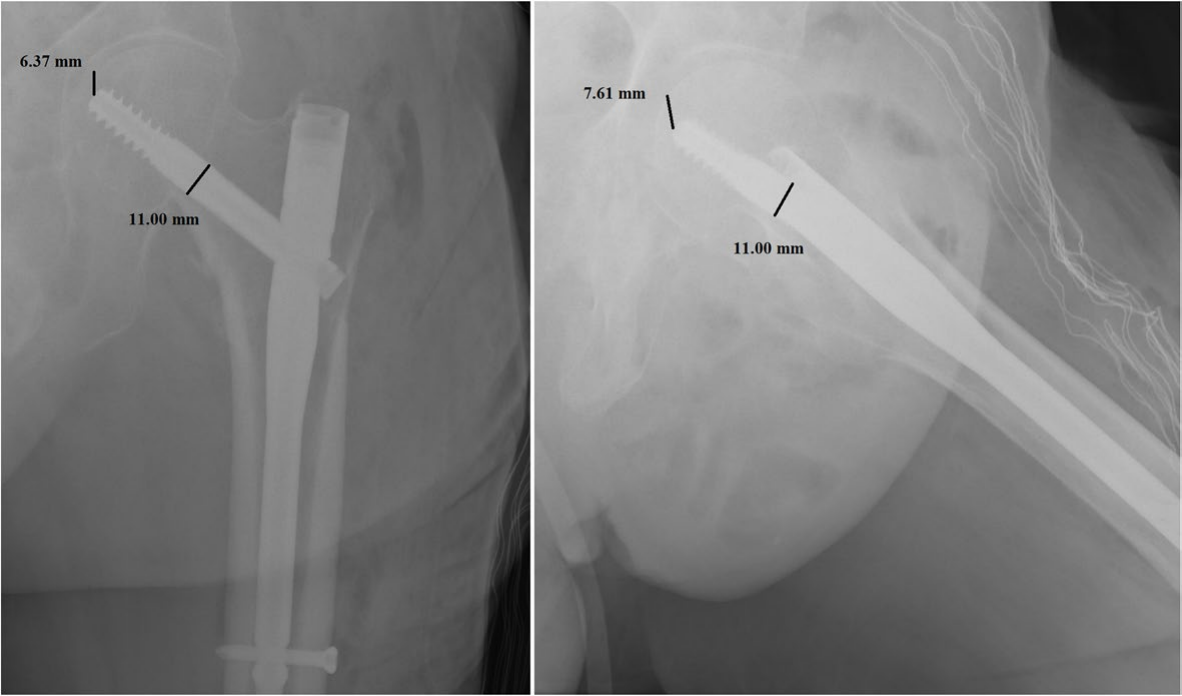
Measurements in antero-posterior and lateral; tip-to-apex distance < 25 mm (Zimmer Natural Nail)

**Fig. 9:**
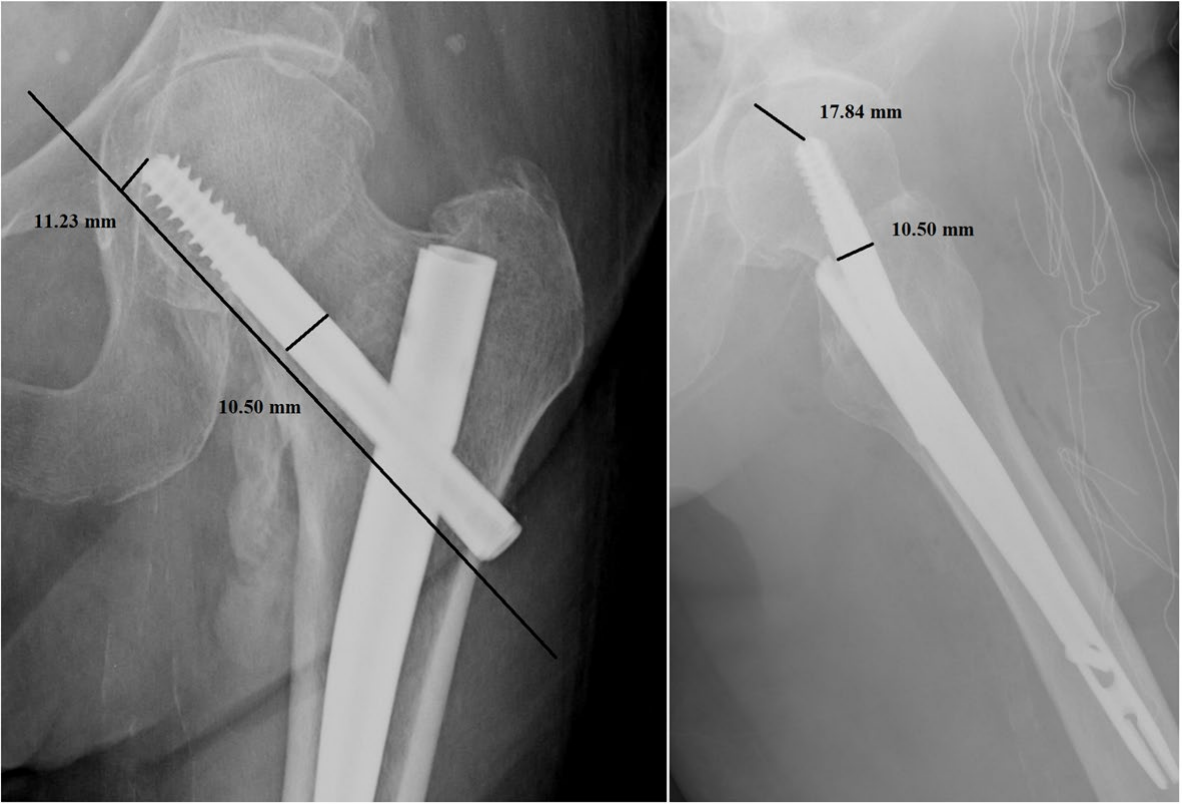
Measurements in antero-posterior and lateral; calcar-referenced tip-to-apex distance > 25 mm (ELOS nail)

In the present investigation, we evaluated the TAD and CalTAD, both valid and reliable predictors of cephalic screw stability. When operating, the correct positioning of the cephalic screw takes place with the aid of an image intensifier, and more than 80% of surgeons who know the concept of TAD are able to position the cephalic screw correctly [14], aiming for TAD and calTAD lower than 25 [14] (Figs. 7, 8, and 9).

Some authors however find discrepancies with these values [22], from anatomical differences and depending on the size of the femoral head, sex, and anthropometric characteristics [23].

The mobilization of the cephalic screw is also a consequence of the poor quality of the bone in which the device is inserted, and patients with greater fragmentation of the fracture have a greater risk of mobilization [14].

A TAD of 25 mm or lower was achieved in a higher percentage of patients treated with the ZNN, as was a CalTAD of 25 mm or lower. However, the comparison between TAD and CalTAD in the ELOS and ZNN implants is affected by the greater number of ZNN implants performed, so this result does not indicate a difference but a similarity between the two devices.

The present work has several strengths. We were able to analyse a relatively large number of patients as our department is a regional referral centre for hip fractures [24]. The patients all followed the same pre- and post-operative therapeutic protocols. The department employs an orthogeriatrician who deals with the management of medical comorbidities [24]. All the procedures were performed by a single orthopaedic surgeon who was fully conversant with the implants, having performed no less than 50 surgeries with both devices prior to the study.

We are aware that a limitation is the lack of randomization to the use of one or the other nail. However, the choice of the IM nail was dictated by their immediate availability, which was independent of the choice of the surgeon. This accounts also for the discrepancy in numbers between the two groups of patients. The present study aimed to evaluate the operative result up to the patients’ discharge, and we have not included information on the follow-up and subsequent radiographic controls. Although the rehabilitation of patients was the same for the different types of devices, data on patients’ compliance are not available. Given our departmental policy and structure, patients were discharged to the care of their general practitioner, who then arranged for urgent orthopaedic follow-up had they deemed it necessary. This is a partial deficiency of the present investigation, as we cannot be certain that patients did not develop a cut-out. However, as the treating centre is the county referral centre for these patients, had they experienced such complications, they would have returned under our care.

Clearly, this is a single-centre single-surgeon study, and the operating surgeon has a special interest and expertise in these injuries. These results need therefore to be validated by larger multicentre studies. We acknowledge that a formal power analysis was not performed: the number of patients enrolled in the study was nevertheless representative for these fractures. However, despite this partial weakness of the present investigation, our selection and recruitment process, our assessment criteria, and data collection were extremely rigorous and performed in strict scientific fashion. Also, with the numbers of patients enrolled, the results of our study are clear. We acknowledge that the study is focused primarily on the length of hospital stay and immediate post-operative outcomes, and not on the short- and long-term clinical effects of the surgery. These limitations may nevertheless affect the reliability of the conclusion; therefore, data must be interpreted with caution. However, despite these limitations, all the surgical procedures were performed in the same fashion and with same instruments, modalities, and materials, resulting in outcomes comparable with other published studies.

## Conclusions

This study evaluates two cephalomedullary devices commonly used in the surgical management of pertrochanteric fractures. In the hands of an experienced surgeon, both devices give similar results in terms of short-term post-operative outcome and hospitalization. The price difference between the devices does not translate in different short-term results on the operated patients.

## Data Availability

The data underlying this article are available in the article and in its online supplementary material.

## Abbreviations

ZNN: Zimmer Natural Nail
SHS: Sliding hip screws
IM: Intramedullary
TAD: Tip-to-apex distance
CalTAD: Calcar-referenced TAD
AP: Antero-posterior
